# Clinical Analytics Prediction Engine (CAPE): Development, electronic health record integration and prospective validation of hospital mortality, 180-day mortality and 30-day readmission risk prediction models

**DOI:** 10.1371/journal.pone.0238065

**Published:** 2020-08-27

**Authors:** Nirav Shah, Chad Konchak, Daniel Chertok, Loretta Au, Alex Kozlov, Urmila Ravichandran, Patrick McNulty, Linning Liao, Kate Steele, Maureen Kharasch, Chris Boyle, Tom Hensing, David Lovinger, Jonathan Birnberg, Anthony Solomonides, Lakshmi Halasyamani

**Affiliations:** 1 NorthShore University HealthSystem, Evanston, Illinois, United States of America; 2 University of Chicago Pritzker School of Medicine, Chicago, Illinois, United States of America; University of Oregon, UNITED STATES

## Abstract

**Background:**

Numerous predictive models in the literature stratify patients by risk of mortality and readmission. Few prediction models have been developed to optimize impact while sustaining sufficient performance.

**Objective:**

We aimed to derive models for hospital mortality, 180-day mortality and 30-day readmission, implement these models within our electronic health record and prospectively validate these models for use across an entire health system.

**Materials & methods:**

We developed, integrated into our electronic health record and prospectively validated three predictive models using logistic regression from data collected from patients 18 to 99 years old who had an inpatient or observation admission at NorthShore University HealthSystem, a four-hospital integrated system in the United States, from January 2012 to September 2018. We analyzed the area under the receiver operating characteristic curve (AUC) for model performance.

**Results:**

Models were derived and validated at three time points: retrospective, prospective at discharge, and prospective at 4 hours after presentation. AUCs of hospital mortality were 0.91, 0.89 and 0.77, respectively. AUCs for 30-day readmission were 0.71, 0.71 and 0.69, respectively. 180-day mortality models were only retrospectively validated with an AUC of 0.85.

**Discussion:**

We were able to retain good model performance while optimizing potential model impact by also valuing model derivation efficiency, usability, sensitivity, generalizability and ability to prescribe timely interventions to reduce underlying risk. Measuring model impact by tying prediction models to interventions that are then rapidly tested will establish a path for meaningful clinical improvement and implementation.

## Introduction

Health care providers and policymakers have identified periods of high clinical intensity and cost of care as important opportunities to improve health care value. Over 25% of all Medicare dollars are spent in the last year of life [1] and roughly 19.8% of Americans die in hospitals [2]. Another period with high clinical intensity and cost is the 30 days after hospital discharge. Each year, approximately 18% of Medicare patients who were discharged were readmitted within 30 days [3], resulting in costs of $15 to 17 billion for unplanned rehospitalizations [4]. Given the increasing trend towards value-based care and penalties for adverse outcomes, hospitals and health systems are actively looking for ways to improve these linked and important outcomes.

Improving care value during these time periods may be based on identification of the highest risk patients. These patients account for a disproportionately high number of adverse outcomes, and are likely to benefit from additional support. Predictive modeling is a commonly used risk stratification strategy. The vast majority of mortality and readmission predictive models focus on maximizing performance of the models rather than real-world impact on care delivery. In doing so, model developers may use predictors that require data collection from additional workflows [5–6], use variables that may not be fully available or have to mature during the hospitalization [6–9], or may not integrate the model into their electronic health record (EHR) either for real-time prospective validation or for calculation to support clinical workflows [6, 10–12].

Furthermore, current predictive models are frequently focused on highly specific segments of the population, such as the elderly [13–14], or on specific disease states, such as pneumonia [11, 15], chronic obstructive pulmonary disease[16, 17] or heart failure [11, 18–21]. While this approach can lead to improved model performance, it tends to cover only a subset of the population, and may not fully recognize that complex patients have multiple, often related, diagnoses that are together driving their clinical risk. By focusing on a narrow patient subset, these types of predictive models are also less likely to improve overall hospital or health system-wide quality or patient safety metrics and are less likely to support broad care transformation that improves outcomes at a population level.

We undertook to design a population health framework, the Clinical Analytics Prediction Engine (CAPE,) for care transformation and to maximize impact across our entire patient population. The initial steps in this development were derivation, retrospective validation, integration of models into our EHR, and prospective validation of three models (hospital mortality, 180-day mortality and 30-day readmission).

The contributions of this work include showing

1. The importance and implications of prospective model validation directly in the EHR.
2. The implications of timing, model performance and patient scoring when predictive models are directly integrated within an EHR.

## Materials and methods

### Settings and participants

Hospital mortality, 180-day mortality and 30-day readmission models were derived and then retrospectively and prospectively validated on data available from patients admitted to NorthShore University HealthSystem (NUHS), Evanston, IL from January 1, 2012, to September 30, 2018. At that time, NUHS was a four-hospital integrated tertiary care health care system. The models were trained on patient data for those admitted on or after January 1, 2012, and discharged before December 31, 2012 (training dataset). The models were also validated on patients admitted on or after December 31, 2012, and discharged on or before April 30, 2017 (retrospective testing dataset) and separately validated with data from May 25, 2018, to September 30, 2018 (prospective testing cohort). Retrospective testing for the 180-day mortality model was restricted to patients discharged before December 30, 2016, to ensure adequate time to capture the outcome. We were unable to prospectively validate the 180-day mortality model due to the lack of mortality data at the time of manuscript preparation.

### Definitions

**Fig 1:**
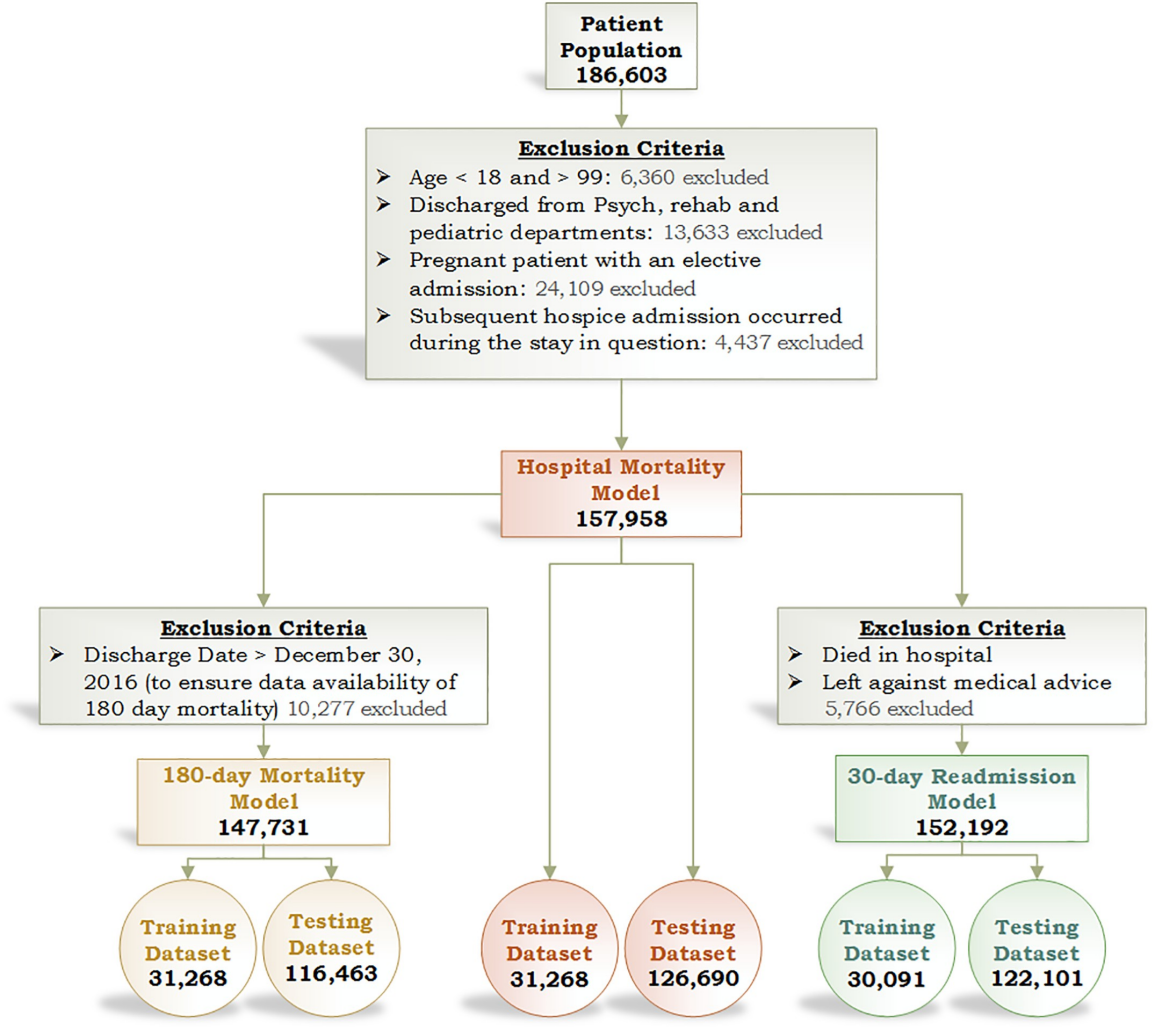
Inclusion and exclusion criteria for derivation and retrospective validation cohorts.

The primary outcomes were mortality during the index admission, mortality within 180 days after discharge from index hospitalization, and all-cause readmission to a NUHS hospital within 30 days after discharge from the index hospitalization. Patients were included in all three models if admission type was inpatient or observation and age was 18 to 99 years old (Fig 1). We excluded psychiatric, rehabilitation and elective pregnancy admissions, as well as patients who had a subsequent hospice admission directly after the index admission. Patients who died during the index hospitalization or left against medical advice were excluded from the 30-day readmission model. We used the same exclusion criteria for the training, retrospective testing and prospective validation datasets.

### Data collection and model derivation

We collected data on demographics, past medical history (PMH), diagnoses present on admission (POA) and labs for all eligible patients for the training, testing and prospective validation datasets from our Enterprise Data Warehouse (EDW). The EDW captures clinical and administrative data during inpatient encounters and also incorporates external data from the Social Security Death Index. All data were collected at the encounter level. Variables were considered for inclusion based on whether they had been previously studied, were consistently reported in the EHR and were collected in real time for risk score computation within the EHR for prospective validation and implementation. Our models did not include social history or social determinants (tobacco and alcohol use, employment, education, living situation and marital status) as these were not consistently reported in our EHR. We identified PMH and POA variables from administrative ICD-9-CM and ICD-10-CM codes associated with the index encounters. We used Clinical Classification Software (CCS) categories to group diagnosis codes to the variables of interest (https://www.hcup-us.ahrq.gov/toolssoftware/ccs/ccs.jsp). Labs were used as continuous variables. Models were derived in R (R Foundation for Statistical Computing, Vienna, Austria; http://www.R-project.org) as logistic regressions for each outcome [22].

X=argmaxL(β), (1)

$$L(\beta )=ln[l(\beta ))=\sum_{i=0}^{N}\{y_{i}\;ln[\pi (X_{i})]+(1-\;y_{i})ln[1-\pi (X_{i})]\},\;$$ (2)

$$\pi (X_{i})=\frac{1}{1+e^{-\sum_{j=0}^{M}\beta_{j}x_{ij}}},\;i=1,\ldots ,N,$$ (3)

where M is the dimensionality of the problem, including the intercept as the 0-th term, x_i0_, N is the number of observations, β = [β_0_ = 1, β_1_, …, β_M_]^T^ is a vector of variable coefficients, y_i_ is the i-th observed outcomes and X_i_ = [x_i0_, …, x_iM_]^T^ is a vector of observed predictor values in the resulting likelihood equations.

$$\sum_{i=1}^{N}{[y}_{i}-\;\pi (X_{i})]=0,$$ (4)

$$\sum_{i=1}^{N}{X_{i}[y}_{i}-\;\pi (X_{i})]=0.$$ (5)

We constructed our variable set by compiling a list of clinically plausible candidate predictors. We imputed sparsely populated binary indicator variables, such as PMH and POA as negatives, e.g., a patient with a missing heart failure indicator would be treated as not having heart failure. We excluded lab variables that were less than 80% populated. Following a common practice of avoiding potential multicollinearity [23], we retained one variable from each pair of variables correlated at 0.6 or higher. While the threshold for such removal is somewhat arbitrary and some practitioners recommend 0.5 or 0.7 as a cutoff [24, 25]; 0.6 has proven more consistent with our experience. We estimated odds ratios and their 95% confidence intervals (CI) for each variable and removed indicator variables whose 95% CI for the univariate odds ratio included 1. For the sake of parsimony, we also excluded indicator variables whose weighted contribution was less than 0.1 and then retrained the models.

### Implementation and validation

In order to partition the datasets for model construction and validation, we opted to reverse the traditional 80% / 20% training and testing split. This was done because available 180-day mortality data from external sources was incomplete after December 31, 2012. Consequently, we opted to derive the model on the data from 2012. Model performance was initially evaluated on the training dataset via 10-fold cross-validation [26]. and retrospectively tested on the data from 2013–2017, treating the absence of information about a death event as a negative outcome. We chose to follow this pattern for other models for the sake of uniformity. We tested our three models retrospectively through the EDW and we validated hospital mortality and 30-day readmission prospectively after direct implementation of our model into our EPIC electronic health record, (EPIC Systems Corporation, Verona, Wisconsin). We intentionally separated the testing dataset where data availability was robust from the prospective validation dataset in order to assess the performance of the models under real-world conditions that mirrored the actual environment in which these models would be deployed.

Integration of our models into the EHR was conducted via Predictive Model Markup Language [27]. Depending on the availability of relevant real-time variables in the EHR, we re-evaluated the models, recalibrated them as necessary using the best available historical proxies for the missing variables and re-implemented the models in the EHR. Non-crucial missing numeric lab values in real-time were zero-imputed in order to differentiate missing values from those that were present. Performance differences between zero- and mean-imputed versions of the same model were trivial for the hospital mortality model, and, based on this finding and operational considerations, we adopted zero imputation for all three predictive models. Prospective validation was performed at two distinct time points, four hours after presentation to the emergency room or hospital floor (Door + 4) and prior to discharge.

### Statistical analysis

All analysis was performed using R. We used Kruskal-Wallis rank sum test on continuous variables and χ^2^ on categorical variables to verify the similarity of populations among the training, testing and prospective validation datasets. We evaluated the performance of the regression models using the AUC and assessed calibration with the Hosmer-Lemeshow statistic. This work was deemed quality improvement after review by our institutional review board (IRB). As such this dataset was coded and not fully anonymized and written consent was not obtained from patients.

## Results

There were 186,603 patient encounters for an inpatient visit between January 1, 2012, and April 30, 2017 (Table 1).

**Table 1:** Patient characteristics in training, testing and prospective validation datasets for hospital mortality model.

Characteristic	Retrospective Training	Retrospective Testing	Prospective Validation	P-value
N (%) or Median	N = 31,268	N = 126,690	N = 11,807	
Age in years	70.6	71.4	71.2	0.8468
Female	17,754 (56.8%)	68,764 (54.3%)	6,436 (54.5%)	0.0000
Male	13,514 (43.2%)	57,926 (45.7%)	5,371 (45.5%)	0.0000
Body Mass Index (kg/m^2^)	25.82	26.58	26.57	0.8296
Pregnant	143 (0.5%)	845 (0.7%)	136 (1.2%)	0.0000
**Ethnicity**				
Hispanic/Latino	1,090 (3.5%)	4,543 (3.6%)	618 (5.2%)	0.0000
Non-Hispanic	30,173 (96.5%)	121,936 (96.2%)	11,145 (94.4%)	0.0000
**Race**				
African American	2,255 (7.2%)	8558 (6.8%)	951 (8.1%)	0.0000
Asian	1,086 (3.5%)	4,661 (3.7%)	555 (4.7%)	0.0000
Caucasian	23,086 (73.8%)	94,880 (74.9%)	8,253 (69.9%)	0.0000
Other	4,841 (15.5%)	18,591 (14.7%)	2,048 (17.3%)	0.0000
**Admission Type**				
Inpatient	23,705 (75.8%)	99,454 (78.5%)	8,541 (72.3%)	0.0000
Observation	7,563 (24.2%)	27,236 (21.5%)	3,266 (27.7%)	0.0000
**Present On Admission**				
Atrial Fibrillation	6,664 (21.3%)	30,292 (23.9%)	365 (3.1%)	0.0000
Chronic Obstructive Pulmonary Disease	3,753 (12.0%)	15,725 (12.4%)	107 (0.9%)	0.0000
Cancer	3,508 (11.2%)	13,604 (10.7%)	72 (0.6%)	0.0000
Injury	2,956 (9.5%)	13,747 (10.9%)	437 (3.7%)	0.0000
Cognitive Disorder	2,932 (9.4%)	11,512 (9.1%)	37 (0.3%)	0.0000
Ventricular Heart Disease	2,904 (9.3%)	11,500 (9.1%)	29 (0.2%)	0.0000
**Medical History**				
Atrial Fibrillation	13,348 (42.7%)	62,169 (49.1%)	6,918 (58.6%)	0.0000
Cancer	11,707 (37.4%)	53,375 (42.1%)	4,611 (39.1%)	0.0000
Pnemonia	8,643 (27.6%)	39,817 (31.4%)	3,457 (29.3%)	0.0000
Ventricular Heart Disease	7,500 (24.0%)	37,308 (29.4%)	3,101 (26.3%)	0.0000
Chronic Obstructive Pulmonary Disease	7,274 (23.3%)	34,615 (27.3%)	3,126 (26.5%)	0.0000
Peripheral Vascular Disease	6,684 (21.4%)	34,717 (27.4%)	3,186 (27.0%)	0.0000
Neurological Condition	6,478 (20.7%)	31,261 (24.7%)	3,140 (26.6%)	0.0000
Syncope	5,055 (16.2%)	22,208 (17.5%)	2,702 (22.9%)	0.0000
Coagulation	4,854 (15.5%)	24,685 (19.5%)	1,529 (12.9%)	0.0000
Cognitive Disorder	4,192 (13.4%)	19,007 (15.0%)	1,507 (12.8%)	0.0000
>3 Comorbid Medical Conditions	16,202 (51.8%)	76,105 (60.1%)	6,762 (57.3%)	0.0000
**Prevalence of outcomes**				
Hospital mortality	590 (1.9%)	2,477 (2.0%)	145 (1.2%)	0.0000
180 day mortality	3,618 (11.6%)	10,097 (8.0%)	-	-
30 day readmission	3,609 (11.5%)	14,034 (11.1%)	1,207 (10.2%)	0.0004
Length of Stay, days	3	3	3	0.5180

When initial exclusion criteria were applied to this population, we had 157,958 patient encounters that met the requirements for the hospital mortality dataset. After additional exclusion criteria were applied, 147,731 encounters remained in the 180-day mortality dataset and 152,192 encounters in the 30-day readmission dataset. These three populations were split into training and testing datasets as detailed in Fig 1. There were 11,807 patients in the prospective validation cohort.

In comparing training, testing and prospective validation datasets for the hospital mortality model, we found that, aside from median age, body mass index (BMI), and length of stay (LOS), the patients in the three populations were statistically significantly different in the proportion of all other risk factors and outcomes. Patients in the traing dataset were on average 70.6 years of age, 56.8% female and 73.8% Caucasian. In the training dataset, hospital mortality was 1.9%, 180-day mortality was 11.6% and 30-day readmission was 11.5%. The prospective validation dataset reflects a population where variables were collected at Door + 4. As a result, POA variables for the prospective validation dataset were significantly more sparsely populated than in the training and testing datasets where those variables were collected retrospectively after maturation.

**Fig 2:**
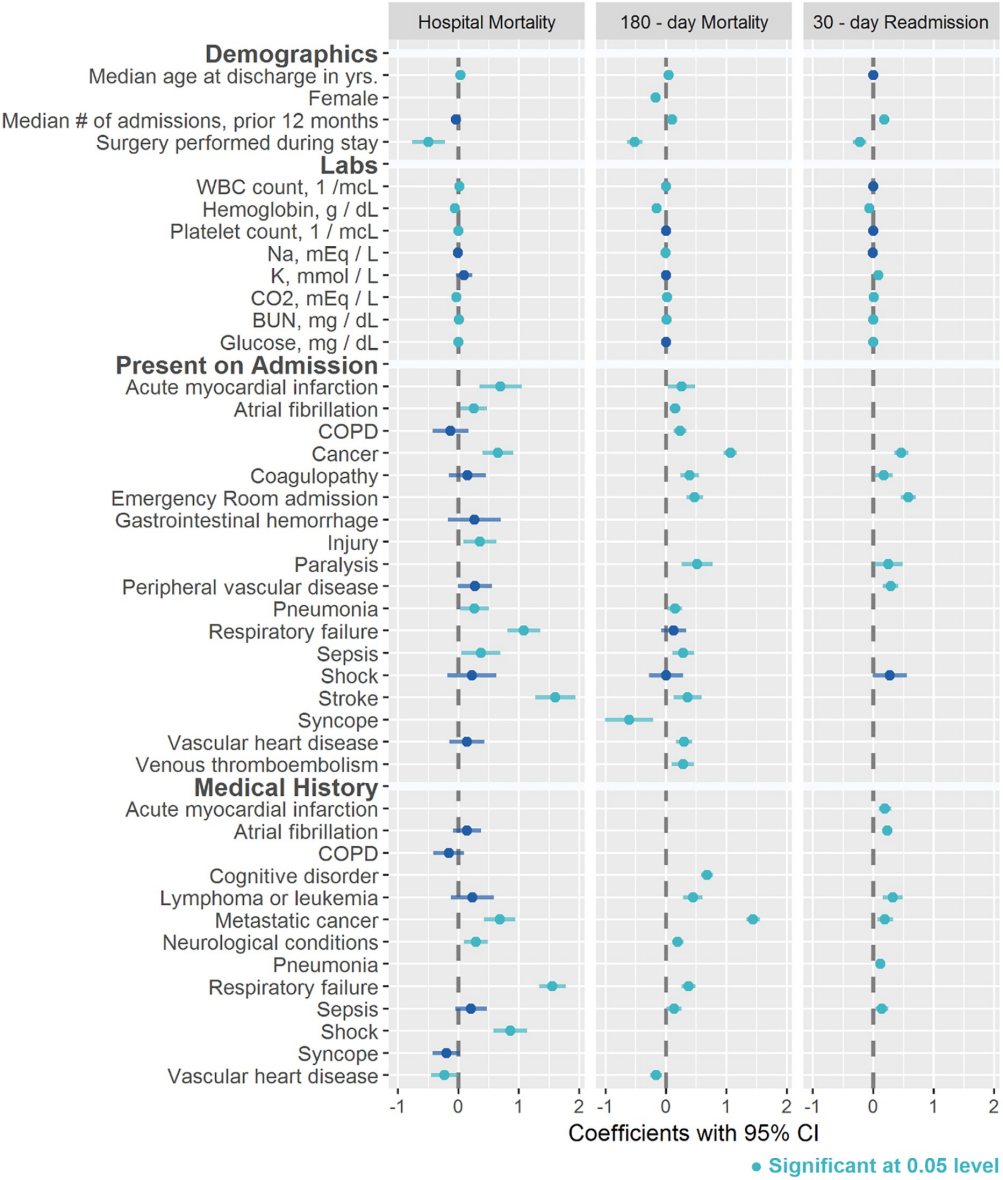
Coefficients and confidence intervals for hospital mortality, 180-day mortality and 30-day readmission prediction models. Abbreviations: WBC, white blood cell; NA, sodium; K, potassium; BUN, blood urea nitrogen; COPD, chronic obstructive pulmonary disease.

Model coefficients and CI for the three predictive models are displayed in Fig 2. Full numerical results of coefficients, CI and odds ratios are included in S1 Table. The variables were grouped into four categories (demographics, labs, POA and PMH) and most of the variables are relevant across all three models. Increasing age was a risk factor for both mortality models but was not significant for 30-day readmission. Female gender is protective in the 180-day mortality model but was not included as a covariate in the final hospital mortality or readmission models. Increasing number of prior admissions increases the risk of 180-day mortality and 30-day readmission but is not significant for the hospital mortality model. Surgery during the index hospitalization is protective for all models. For the hospital mortality model, the variables most predictive of death within the POA domain were stroke and respiratory failure; respiratory failure dominated the risk for the PMH domain. For 180-day mortality, the variable most predictive of death within the POA domain was cancer; within the PMH domain, it was metastatic cancer. Admission to the hospital from the emergency room and a POA cancer diagnosis were the most predictive factors of 30-day readmission within the POA domain. The 30-day readmission model had fewer POA covariates present in the model as compared to the mortality models.

Coefficients in the plot are not standardized for interpretability. Pregnancy on admission was an outlier in the 180-day Mortality model (-10.9993 [95% CI -241.7027: 219.7041], p-value 0.9255) due to the rarity of the condition and while it was a covariate in the model it was not included in Fig 2. Pregnancy on admission was not a covariate for Hospital Mortality and 30-day Readmission models.

Using 10-fold cross validation within the training dataset, the AUC of the hospital mortality model was 0.90 (95% CI 0.87–0.93), 180-day mortality model was 0.85 (95% CI 0.84–0.87) and 30-day readmission was 0.71 (95% CI 0.69–0.73). In all three models, the CI of the cross-validated performance was tight, and the coefficients were stable.

**Fig 3:**
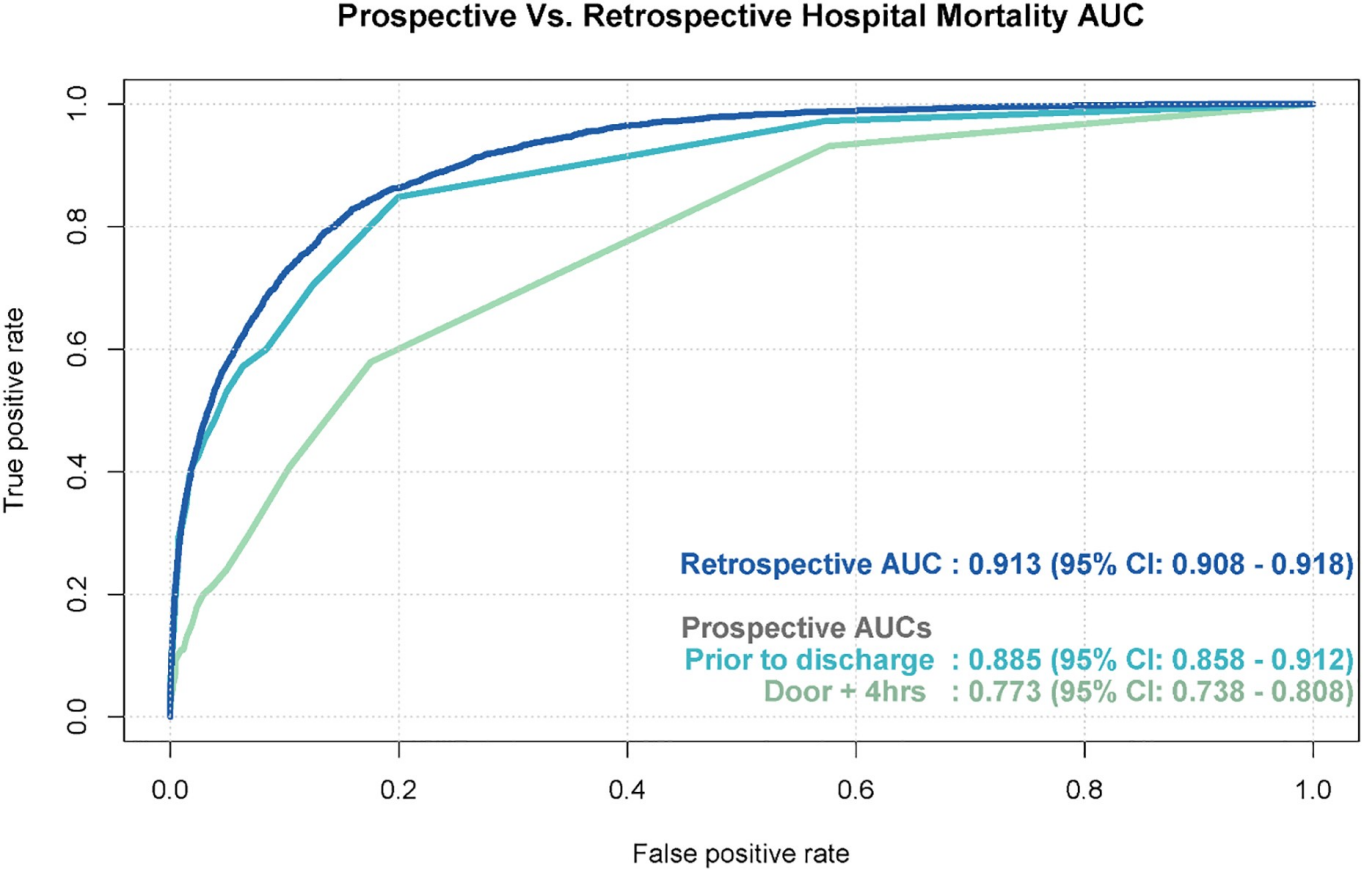
Hospital mortality model performance for retrospective and prospective validation.

**Fig 4:**
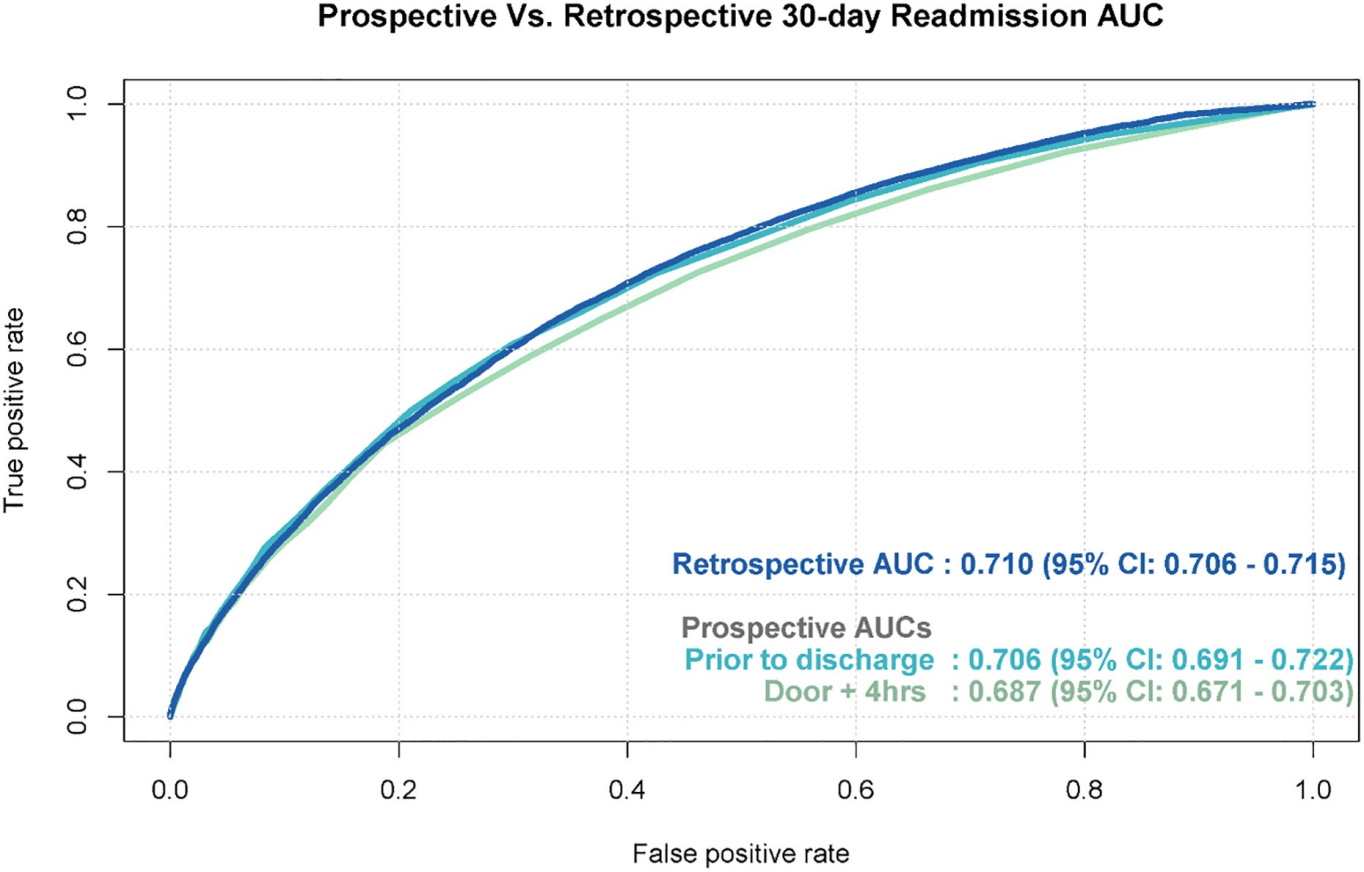
30-day readmission model performance for retrospective and prospective validation.

Performance of the models was measured on the testing dataset by the AUC at three time points: retrospective, prospective at discharge, and prospective at Door + 4 (Figs 3 and 4). AUCs of the hospital mortality prediction model were 0.91, 0.89 and 0.77, respectively. The 180-day mortality model yielded the AUC of 0.85 retrospectively; accurate prospective 180-day mortality data was not available at time of manuscript preparation and, as a result, prospective validation was not performed on this model. The AUC of the 30-day readmission model was 0.71 retrospectively and prospectively prior to discharge and 0.69 at Door + 4. Performance degradation occured from the retrospective to prospective period for both hospital mortality and 30-day readmission models, but performance improved from Door + 4 to the time of discharge. The hospital mortality model showed satisfactory calibration (Hosmer Lemeshow p = 0.0418) as did the 30-day readmission model (Hosmer Lemeshow p<0.001) with the prospective dataset. Matthew’s correlation coefficient and F1 accuracy measures are included in S1 and S2 Figs.

We calculated the risk of hospital mortality, 180-day mortality and 30-day readmission at two different time points within our prospective validation dataset in order to understand how patient risk scores may change throughout their hospital stay (Tables 2–4). We designated patients as high risk if their scores were in the top 10% for hospital mortality, top 25% for 180-day mortality, or top 25% for 30-day readmission. We used this threshold designation primarily for operational purposes (not discussed in this work) to evaluate a combined high risk state. The vast majority of patients retained their risk categorization from Door + 4 to the time at discharge. with fewer than 10% of the population migrating between risk groups. Patient risk was more likely to increase than to decrease throughout hospital stay.

**Table 2:** Hospital mortality risk scoring at Door + 4 and prior to discharge.

Hospital Mortality (N = 11,807)		Prior to Discharge	
		High	Low
**Door + 4**	**High**	1,857 (15.73%)	274 (2.32%)
	**Low**	592 (5.01%)	9,084 (76.94%)

**Table 3:** 180-day mortality risk scoring at Door + 4 and prior to discharge.

180 Day Mortality (N = 11,807)		Prior to Discharge	
		High	Low
**Door + 4**	**High**	3425 (29.01%)	325(2.75%)
	**Low**	616 (5.22%)	7441 (63.02%)

**Table 4:** 30-day readmission risk scoring at Door + 4 and prior to discharge.

30 Day Readmission (N = 11,807))		Prior to Discharge	
		High	Low
**Door + 4**	**High**	3,771 (31.94%)	323 (2.74%)
	**Low**	854 (7.23%)	6,859 (58.09%)

## Discussion

We describe the development of three health care system-wide predictive models (hospital mortality, 180-day mortality and 30-day all-cause readmission), their retrospective testing and integration directly into our electronic health record, and the subsequent prospective validation of two of these models at different time points within a patient’s hospitalization. Our EHR-embedded models were tested both retrospectively and prospectively, so that knowledge of subsequent history could not contaminate the results. All exhibit good performance in retrospective and real-time prospective validation and are able to provide risk scores for patients at different time points throughout their hospital stay.

In an effort to operationalize the models, we adopted the score calculated as close as possible to the 4-hour mark after the initial presentation into the emergency department or directly to the hospital floor. This timing would allow us to provide the care team with an actionable risk assessment of the patient’s state at the expense of sacrificing some model accuracy. Attention to the requirement of usability and actionability of the model for care providers outweighed small improvements in model performance. The resulting drop in model AUC, compared to the best available post hoc data, was trivial for the readmission model and acceptable for the hospital mortality model. Data availability through the course of the hospitalization (primarily labs and refinement of diagnostic documentation) is responsible for changes in performance. These changes represent a very practical challenge in using predictive models: the true performance of a model should be judged when the output is being used to impact care in real time. Despite the emphasis on actionability and usability, our hospital mortality and readmission models performed as well as similar models in the literature (AUC range 0.7–0.88) [6–7, 11, 28], where, in general, direct EHR model implementations are limited (see S2 Table in online supplement)Compared to some studies using deep learning on large rich datasets [10], we have traded a small reduction in performance for responsive development of effective parsimonious models, noting that current EHR systems are limited in the types of advanced modeling that can be implemented, in particular with PMML.

One exception of direct EHR model implementation is included in a recent study from the Netherlands, where investigators present a model developed and prospectively validated to determine the likelihood of admission from the emergency department. This was subsequently integrated into the EHR to assist with future work in triage [29]. Our work differs with the novel feature that we integrated our models into the EHR and undertook prospective validation, so that the entire system, EHR and model together, were evaluated.

This work shares with models in the existing literature an efficient process of predictive model development that allows for risk stratification and patient segmentation for three simultaneous key outcomes applicable to the vast majority of patients in a health care system [10]. This integrated approach is potentially more impactful than the traditional approach commonly found in the literature that focuses on targeting outcomes for specific clinical conditions or populations. While this singular method improves model performance it considerably limits the population on which one would like to make an impact. In future work, we are working towards integrated and holistic set of interventions tied to these models which will allow for determination of full model impact.

Our models and corresponding findings add to the current literature in two novel ways. First, we integrated our models directly into the EHR and performed prospective validation without a significant degradation in performance. This latter step is crucial in assuring adequate model performance at the point of care, which is an absolute necessity when applying these models to improve population health outcomes. It is imperative that scores be based on real-time variables that evolve as the admission progresses, not only on historical inputs that may have been derived from fully collected and mature data obtained on or after discharge. Furthermore, the variables chosen were readily available within our EHR and did not require additional workflow for capture, which allows this approach to be replicated by other health systems. The latter requirement provided a feedback loop into another iteration of model development since a real-time version of the model could only include stable high quality data available as early as possible after admission for the initial score. This requirement dictated the need to discard potential predictors that only fully matured at a much later point during the patient’s stay. We prioritized the inclusion of a more limited but timely set of inputs to a full set of candidate variables so that the model could be deployed in clinical care delivery. Despite this tradeoff, it was able to perform as well as similar models in the literature.

Second, we were able to demonstrate how model performance and risk scoring vary based on the time at which risk is calculated during the index hospitalization. Variable model performance over time has significant implications on the timing of workflows and the aligning of resources to perform prescribed interventions in order to achieve an impact on the predicted outcome We are currently working towards an integrated and holistic set of interventions tied to these models. This will allow further calibration of the model as well as a more accurate determination of model impact. By evaluating model performance at different times, we introduce new considerations when evaluating hospital-initiated population health initiatives that prescribe interventions based on predictive modeling. Risk scoring, like model performance, may also vary at different time points during a hospitalization. Risk can be reclassified when modifiable clinical factors (covariates), such as laboratory values, changes in and new data, such as revised diagnoses, become available. For a small subset of patients where risk scoring is reclassified, improving early data availability may be impactful in reducing misclassification. The implications of timing, model performance, risk scoring and misclassification are important considerations and warrant further study.

There were limitations to this work. The data were collected from a single health system, which may limit generalizability. This shortcoming is mitigated by the fact that our health system consists of four distinct hospitals. The models retained good performance in retrospective testing and prospective validation, even though most patient characteristics in the training, testing and prospective (Door + 4) validation datasets were statistically significantly different from each other (Table 1), speaking to the potential generalizability of the models and the ability of our model to perform well during the early stages of a hospitalization when some data elements are not yet mature. Our prospective validation period was just over four months, which may not have provided adequate time to capture the outcomes of interest in sufficient numbers or fully account for seasonality, potentially resulting in the degradation of prospective performance. Additionally, we excluded patients who were discharged to hospice from the models. We excluded this patient population in part because it was not clear when during the hospitalization they were enrolled into hospice, but in any case we tolerated this approach since this population represented 0.46% of the training set.

## Conclusions

Model performance has received significant attention in the literature without adequate consideration of meaningful and timely model impact. Consequently, we propose that it is important to consider model impact and model performance together. If the goal is model implementation to improve clinical outcomes, then it is important to prioritize model impact even if there are modest consequences for model performance. Model impact involves optimizing model derivation efficiency, performance, usability, sensitivity, generalizability and ability to prescribe timely interventions to reduce underlying risk. Future work will involve measuring model impact by tying scoring of these three prediction models directly to prescribed interventions and rapidly testing out whether these interventions improve outcomes.

## Supporting information

S1 Fig Mathew’s correlation coefficiant. (TIF)

S2 Fig F1 scores. (TIF)

S1 Table Coefficients, odds ratios, confidence intervals and statistical significance of hospital mortality (IH), 180-day mortality (OOH) and 30-day readmission (R) models. (DOCX)

S2 Table Performance comparison. (DOCX)
