# Supplementary figures and images for: Clinical Analytics Prediction Engine (CAPE): Development, electronic health record integration and prospective validation of hospital mortality, 180-day mortality and 30-day readmission risk prediction models
Source: PLoS One. 2020 Aug 27;15(8):e0238065. doi: 10.1371/journal.pone.0238065 (PMC7451512; doi:10.1371/journal.pone.0238065)

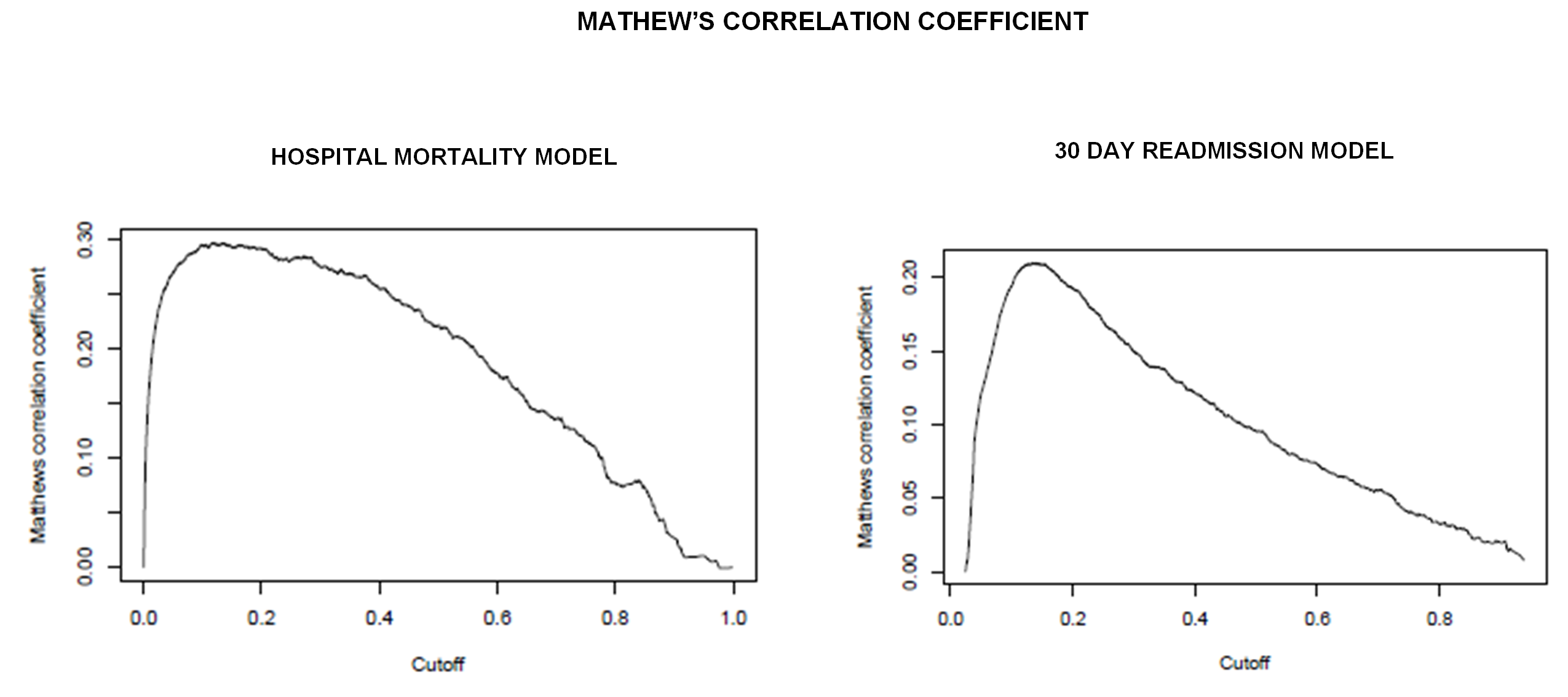

Supplement: S1 Fig — (TIF) [file pone.0238065.s001.tif]

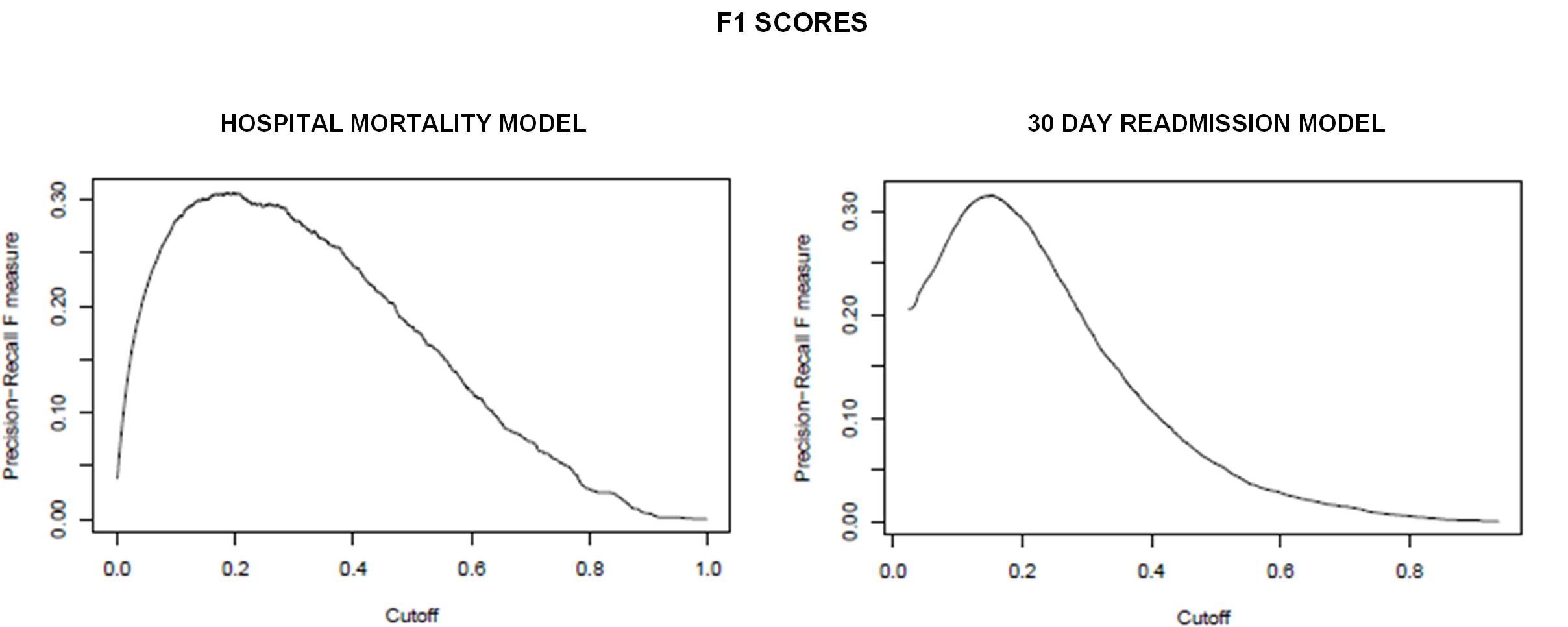

Supplement: S2 Fig — (TIF) [file pone.0238065.s002.tif]
