# Supplementary material for: Clinical Analytics Prediction Engine (CAPE): Development, electronic health record integration and prospective validation of hospital mortality, 180-day mortality and 30-day readmission risk prediction models
Source: PLoS One. 2020 Aug 27;15(8):e0238065. doi: 10.1371/journal.pone.0238065 (PMC7451512; doi:10.1371/journal.pone.0238065)
Supplement: S1 Table — (DOCX) [file pone.0238065.s003.docx]

| **Variable** | **Model** | **Coefficient (CI)** | **p-value** | **Odds Ratio (CI)** | | **p-value** |
| --- | --- | --- | --- | --- | --- | --- |
| **Intercept** | IH | -4.4177 (-6.9605 : -1.8749) | 0.0007 |  | |  |
|  | OOH | -4.1402 (-5.4095 : -2.8709) | 0.0000 |  | |  |
|  | R | -2.091 (-3.3338 : -0.8482) | 0.0010 |  | |  |
| *Patient characteristics* | | |  |  | |  |
| **Median age at discharge** | IH | 0.0313 (0.0238 : 0.0388) | 0.0000 | 1.0318 (1.0253 : 1.0383) | | 0.0000 |
| **in yrs.** | OOH | 0.0405 (0.0370 : 0.0440) | 0.0000 | 1.0413 (1.0382 : 1.0443) | | 0.0000 |
|  | R | 0.0016 (-0.0008 : 0.0040) | 0.1900 | 1.0016 (0.9996 : 1.0036) | | 0.1900 |
| **Female** | IH |  |  |  | |  |
|  | OOH | -0.1672 (-0.2488 : -0.0856) | 0.0001 | 0.846 (0.7901 : 0.9060) | | 0.0001 |
|  | R |  |  |  | |  |
| **Median # of admissions,** | IH | -0.0419 (-0.0889 : 0.0051) | 0.0805 | 0.959 (0.9219 : 0.9975) | | 0.0805 |
| **prior 12 months** | OOH | 0.0993 (0.0830 : 0.1156) | 0.0000 | 1.1044 (1.0894 : 1.1196) | | 0.0000 |
|  | R | 0.1792 (0.1631 : 0.1953) | 0.0000 | 1.1962 (1.1802 : 1.2125) | | 0.0000 |
| **Surgery performed during** | IH | -0.5002 (-0.7721 : -0.2283) | 0.0003 | 0.6064 (0.4827 : 0.7619) | | 0.0003 |
| **stay** | OOH | -0.5158 (-0.6457 : -0.3859) | 0.0000 | 0.597 (0.5354 : 0.6657) | | 0.0000 |
|  | R | -0.2261 (-0.3376 : -0.1146) | 0.0001 | 0.7977 (0.7264 : 0.8759) | | 0.0001 |
| *Labs* |  |  |  |  | |  |
| **BUN, mg / dL** | IH | 0.0098 (0.0058 : 0.0138) | 0.0000 | 1.0098 (1.0064 : 1.0133) | | 0.0000 |
|  | OOH | 0.0097 (0.0076 : 0.0118) | 0.0000 | 1.0097 (1.0079 : 1.0115) | | 0.0000 |
|  | R | 0.0026 (0.0004 : 0.0048) | 0.0205 | 1.0026 (1.0007 : 1.0044) | | 0.0205 |
| **CO2, mEq / L** | IH | -0.0391 (-0.0585 : -0.0197) | 0.0001 | 0.9617 (0.9461 : 0.9775) | | 0.0001 |
|  | OOH | 0.0226 (0.0130 : 0.0322) | 0.0000 | 1.0228 (1.0146 : 1.0311) | | 0.0000 |
|  | R | 0.011 (0.0016 : 0.0204) | 0.0215 | 1.0111 (1.0031 : 1.0191) | | 0.0215 |
| **Glucose, mg / dL** | IH | 0.0013 (0.0002 : 0.0024) | 0.0208 | 1.0013 (1.0004 : 1.0023) | | 0.0208 |
|  | OOH | 0.0004 (-0.0003 : 0.0011) | 0.2656 | 1.0004 (0.9998 : 1.0009) | | 0.2656 |
|  | R | 0.0006 (0.0000 : 0.0012) | 0.0492 | 1.0006 (1.0001 : 1.0011) | | 0.0492 |
| **Hemoglobin, g / dL** | IH | -0.0611 (-0.1050 : -0.0172) | 0.0063 | 0.9407 (0.9067 : 0.9760) | | 0.0063 |
|  | OOH | -0.1522 (-0.1722 : -0.1322) | 0.0000 | 0.8588 (0.8445 : 0.8733) | | 0.0000 |
|  | R | -0.0646 (-0.0832 : -0.0460) | 0.0000 | 0.9375 (0.9229 : 0.9522) | | 0.0000 |
| **Platelet count, 1 / mcL** | IH | -0.0015 (-0.0024 : -0.0006) | 0.0022 | 0.9985 (0.9977 : 0.9993) | | 0.0022 |
|  | OOH | 0.0003 (-0.0001 : 0.0007) | 0.0880 | 1.0003 (1.0000 : 1.0007) | | 0.0880 |
|  | R | 0.0003 (-0.0001 : 0.0007) | 0.1323 | 1.0003 (1.0000 : 1.0006) | | 0.1323 |
| **K, mmol / L** | IH | 0.0923 (-0.0414 : 0.2260) | 0.1759 | 1.0967 (0.9803 : 1.2269) | | 0.1759 |
|  | OOH | 0.0048 (-0.0618 : 0.0714) | 0.8871 | 1.0048 (0.9502 : 1.0626) | | 0.8871 |
|  | R | 0.0835 (0.0193 : 0.1477) | 0.0108 | 1.0871 (1.0301 : 1.1473) | | 0.0108 |
| **Na, mEq / L** | IH | -0.0147 (-0.0318 : 0.0024) | 0.0928 | 0.9854 (0.9714 : 0.9997) | | 0.0928 |
|  | OOH | -0.0099 (-0.0182 : -0.0016) | 0.0186 | 0.9901 (0.9832 : 0.9970) | | 0.0186 |
|  | R | -0.0078 (-0.0161 : 0.0005) | 0.0666 | 0.9923 (0.9854 : 0.9992) | | 0.0666 |
| **WBC count, 1 /mcL** | IH | 0.014 (0.0078 : 0.0202) | 0.0000 | 1.0141 (1.0088 : 1.0194) | | 0.0000 |
|  | OOH | 0.0063 (0.0016 : 0.0110) | 0.0076 | 1.0064 (1.0024 : 1.0103) | | 0.0076 |
|  | R | 0.0006 (-0.0046 : 0.0058) | 0.8071 | 1.0006 (0.9963 : 1.0050) | | 0.8071 |
| *Present on Admission* |  |  |  |  | |  |
| **Emergency Room admission** | IH |  |  |  | |  |
|  | OOH | 0.4767 (0.3374 : 0.6160) | 0.0000 | 1.6107 (1.4330 : 1.8105) | | 0.0000 |
|  | R | 0.5789 (0.4557 : 0.7021) | 0.0000 | 1.784 (1.6088 : 1.9784) | | 0.0000 |
| **Atrial fibrillation** | IH | 0.2503 (0.0286 : 0.4720) | 0.0269 | 1.2844 (1.0663 : 1.5470) | | 0.0269 |
|  | OOH | 0.152 (0.0622 : 0.2418) | 0.0009 | 1.1642 (1.0797 : 1.2553) | | 0.0009 |
|  | R |  |  |  | |  |
| **Acute myocardial infarction** | IH | 0.6943 (0.3413 : 1.0473) | 0.0001 | 2.0023 (1.4889 : 2.6927) | | 0.0001 |
|  | OOH | 0.2593 (0.0395 : 0.4791) | 0.0208 | 1.296 (1.0776 : 1.5586) | | 0.0208 |
|  | R |  |  |  | |  |
| **Cancer** | IH | 0.6519 (0.3944 : 0.9094) | 0.0000 | 1.9192 (1.5463 : 2.3821) | | 0.0000 |
|  | OOH | 1.0649 (0.9525 : 1.1773) | 0.0000 | 2.9004 (2.6394 : 3.1873) | | 0.0000 |
|  | R | 0.4606 (0.3437 : 0.5775) | 0.0000 | 1.585 (1.4369 : 1.7484) | | 0.0000 |
| **Coagulopathy** | IH | 0.1441 (-0.1633 : 0.4515) | 0.3583 | 1.155 (0.8924 : 1.4949) | | 0.3583 |
|  | OOH | 0.3914 (0.2388 : 0.5440) | 0.0000 | 1.479 (1.3012 : 1.6810) | | 0.0000 |
|  | R | 0.173 (0.0205 : 0.3255) | 0.0261 | 1.1889 (1.0461 : 1.3512) | | 0.0261 |
| **COPD** | IH | -0.1351 (-0.4351 : 0.1649) | 0.3774 | 0.8736 (0.6792 : 1.1237) | | 0.3774 |
|  | OOH | 0.232 (0.1256 : 0.3384) | 0.0000 | 1.2611 (1.1534 : 1.3789) | | 0.0000 |
|  | R |  |  |  | |  |
| **Gastrointestinal hemorrhage** | IH | 0.264 (-0.1730 : 0.7010) | 0.2363 | 1.3022 (0.9024 : 1.8790) | | 0.2363 |
|  | OOH |  |  |  | |  |
|  | R |  |  |  | |  |
| **Injury** | IH | 0.3513 (0.0772 : 0.6254) | 0.0120 | 1.4209 (1.1289 : 1.7884) | | 0.0120 |
|  | OOH |  |  |  | |  |
|  | R |  |  |  | |  |
| **Pneumonia** | IH | 0.2645 (0.0272 : 0.5018) | 0.0289 | 1.3028 (1.0676 : 1.5899) | | 0.0289 |
|  | OOH | 0.1474 (0.0286 : 0.2662) | 0.0151 | 1.1588 (1.0488 : 1.2803) | | 0.0151 |
|  | R |  |  |  | |  |
| **Peripheral vascular disease** | IH | 0.2713 (-0.0113 : 0.5539) | 0.0599 | 1.3117 (1.0347 : 1.6628) | | 0.0599 |
|  | OOH |  |  |  | |  |
|  | R | 0.2871 (0.1602 : 0.4140) | 0.0000 | 1.3326 (1.1980 : 1.4824) | | 0.0000 |
| **Respiratory failure** | IH | 1.0826 (0.8106 : 1.3546) | 0.0000 | 2.9524 (2.3498 : 3.7096) | | 0.0000 |
|  | OOH | 0.1288 (-0.0789 : 0.3365) | 0.2244 | 1.1374 (0.9555 : 1.3540) | | 0.2244 |
|  | R |  |  |  | |  |
| **Sepsis** | IH | 0.3676 (0.0445 : 0.6907) | 0.0257 | 1.4443 (1.1013 : 1.8941) | | 0.0257 |
|  | OOH | 0.2873 (0.1104 : 0.4642) | 0.0015 | 1.3328 (1.1489 : 1.5462) | | 0.0015 |
|  | R |  |  |  | |  |
| **Shock** | IH | 0.2214 (-0.1829 : 0.6257) | 0.2831 | 1.2478 (0.8888 : 1.7518) | | 0.2831 |
|  | OOH | 0.0049 (-0.2758 : 0.2856) | 0.9726 | 1.0049 (0.7940 : 1.2719) | | 0.9726 |
|  | R | 0.2722 (-0.0091 : 0.5535) | 0.0579 | 1.3128 (1.0368 : 1.6623) | | 0.0579 |
| **Stroke** | IH | 1.6042 (1.2682 : 1.9402) | 0.0000 | 4.974 (3.7519 : 6.5942) | | 0.0000 |
|  | OOH | 0.3545 (0.1229 : 0.5861) | 0.0027 | 1.4255 (1.1737 : 1.7313) | | 0.0027 |
|  | R |  |  |  | |  |
| **Vascular heart disease** | IH | 0.1367 (-0.1508 : 0.4242) | 0.3515 | 1.1465 (0.9007 : 1.4593) | | 0.3515 |
|  | OOH | 0.2991 (0.1665 : 0.4317) | 0.0000 | 1.3487 (1.2066 : 1.5074) | | 0.0000 |
|  | R |  |  |  | |  |
| **Paralysis** | IH |  |  |  | |  |
|  | OOH | 0.5166 (0.2587 : 0.7745) | 0.0001 | 1.6763 (1.3500 : 2.0814) | | 0.0001 |
|  | R | 0.2505 (0.0106 : 0.4904) | 0.0407 | 1.2847 (1.0504 : 1.5712) | | 0.0407 |
| **Syncope** | IH |  |  |  | |  |
|  | OOH | -0.6093 (-1.0099 : -0.2087) | 0.0029 | 0.5437 (0.3885 : 0.7610) | | 0.0029 |
|  | R |  |  |  | |  |
| **Venous thromboembolism** | IH |  |  |  | |  |
|  | OOH | 0.2795 (0.0907 : 0.4683) | 0.0037 | 1.3225 (1.1288 : 1.5496) | | 0.0037 |
|  | R |  |  |  | |  |
| **Pregnancy** | IH |  |  |  | |  |
|  | OOH | -10.9993 (-241.7027 : 219.7041) | 0.9255 | 0.0000 (0.0000 : Inf) | | 0.9255 |
|  | R |  |  |  | |  |
| *Comorbidities in medical history* | | |  |  | |  |
| **Acute myocardial infarction** | IH |  |  |  | |  |
|  | OOH |  |  |  | |  |
|  | R | 0.19 (0.0861 : 0.2939) | 0.0003 | 1.2092 (1.1082 : 1.3194) | | 0.0003 |
| **Atrial fibrillation** | IH | 0.1385 (-0.0963 : 0.3733) | 0.2478 | 1.1485 (0.9431 : 1.3986) | | 0.2478 |
|  | OOH |  |  |  | |  |
|  | R | 0.2302 (0.1512 : 0.3092) | 0.0000 | 1.2589 (1.1782 : 1.3452) | | 0.0000 |
| **Cognitive disorder** | IH |  |  |  | |  |
|  | OOH | 0.6821 (0.5833 : 0.7809) | 0.0000 | 1.9781 (1.8207 : 2.1490) | | 0.0000 |
|  | R |  |  |  | |  |
| **COPD** | IH | -0.1622 (-0.4132 : 0.0888) | 0.2055 | 0.8503 (0.6888 : 1.0497) | | 0.2055 |
|  | OOH |  |  |  | |  |
|  | R |  |  |  | |  |
| **Lymphoma or leukemia** | IH | 0.2301 (-0.1244 : 0.5846) | 0.2033 | 1.2587 (0.9348 : 1.6948) | | 0.2033 |
|  | OOH | 0.4452 (0.2851 : 0.6053) | 0.0000 | 1.5608 (1.3646 : 1.7852) | | 0.0000 |
|  | R | 0.3174 (0.1520 : 0.4828) | 0.0002 | 1.3735 (1.1954 : 1.5781) | | 0.0002 |
| **Metastatic cancer** | IH | 0.6798 (0.4208 : 0.9388) | 0.0000 | 1.9735 (1.5880 : 2.4526) | | 0.0000 |
|  | OOH | 1.4424 (1.3319 : 1.5529) | 0.0000 | 4.2307 (3.8561 : 4.6416) | | 0.0000 |
|  | R | 0.1912 (0.0693 : 0.331) | 0.0021 | 1.2107 (1.0930 : 1.3411) | | 0.0021 |
| **Neurological conditions** | IH | 0.2848 (0.0875 : 0.4821) | 0.0047 | 1.3295 (1.1266 : 1.5690) | | 0.0047 |
|  | OOH | 0.1956 (0.1032 : 0.2880) | 0.0000 | 1.2161 (1.1254 : 1.3141) | | 0.0000 |
|  | R |  |  |  | |  |
| **Pneumonia** | IH |  |  |  | |  |
|  | OOH |  |  |  | |  |
|  | R | 0.1129 (0.0297 : 0.1961) | 0.0078 | 1.1195 (1.0440 : 1.2004) | | 0.0078 |
| **Respiratory failure** | IH | 1.5555 (1.3337 : 1.7773) | 0.0000 | 4.7376 (3.9331 : 5.7066) | | 0.0000 |
|  | OOH | 0.3722 (0.2547 : 0.4897) | 0.0000 | 1.4509 (1.3147 : 1.6012) | | 0.0000 |
|  | R |  |  |  | |  |
| **Sepsis** | IH | 0.2055 (-0.0545 : 0.4655) | 0.1213 | 1.2282 (0.9874 : 1.5276) | | 0.1213 |
|  | OOH | 0.1383 (0.0144 : 0.2622) | 0.0287 | 1.1484 (1.0349 : 1.2742) | | 0.0287 |
|  | R | 0.1407 (0.0328 : 0.2486) | 0.0106 | 1.1511 (1.0514 : 1.2601) | | 0.0106 |
| **Shock** | IH | 0.8604 (0.5802 : 1.1406) | 0.0000 | 2.3641 (1.8688 : 2.9908) | | 0.0000 |
|  | OOH |  |  |  | |  |
|  | R |  |  |  | |  |
| **Syncope** | IH | -0.2026 (-0.4314 : 0.0262) | 0.0826 | 0.8166 (0.6739 : 0.9894) | | 0.0826 |
|  | OOH |  |  |  |  |  |
|  | R |  |  |  | |  |
| **Vascular heart disease** | IH | -0.233 (-0.4581 : -0.0079) | 0.0426 | 0.7922 (0.6558 : 0.9569) | | 0.0426 |
|  | OOH | -0.1619 (-0.2631 : -0.0607) | 0.0017 | 0.8506 (0.7813 : 0.9259) | | 0.0017 |
|  | R |  |  |  | |  |
