# Supplementary material for: Clinical Analytics Prediction Engine (CAPE): Development, electronic health record integration and prospective validation of hospital mortality, 180-day mortality and 30-day readmission risk prediction models
Source: PLoS One. 2020 Aug 27;15(8):e0238065. doi: 10.1371/journal.pone.0238065 (PMC7451512; doi:10.1371/journal.pone.0238065)
Supplement: S2 Table — (DOCX) [file pone.0238065.s004.docx]

**Supporting Table 2. Performance Comparison**

|  | **Lehn et al.** | **Cowen et al** | **Hebert et al** | **Rajkomar et al** | **Retrospective Shah et al** | **Prospective Shah et al** |
| --- | --- | --- | --- | --- | --- | --- |
| **Readmission model AUC (95% Confidence Interval)** | 0.7 | NA | 0.73-0.76 | 0.75 - 0.76 | 0.71 (0.71 - 0.72) | 0.71 (0.71 - 0.72) |
| **In-hospital mortality model AUC (95% Confidence Interval)** | NA | 0.88 | NA | 0.93 - 0.94 | 0.91 (0.91 - 0.92) | 0.89 (0.86 -0.91) |
